# Supplementary material for: Indirect Negative Effect of Mutant Ataxin-1 on Short- and Long-Term Synaptic Plasticity in Mouse Models of Spinocerebellar Ataxia Type 1
Source: Cells. 2022 Jul 20;11(14):2247. doi: 10.3390/cells11142247 (PMC9317252; doi:10.3390/cells11142247)
Supplement: Supplementary file 1 [file cells-11-02247-s001.zip › cells-1809764-supplementary.pdf]

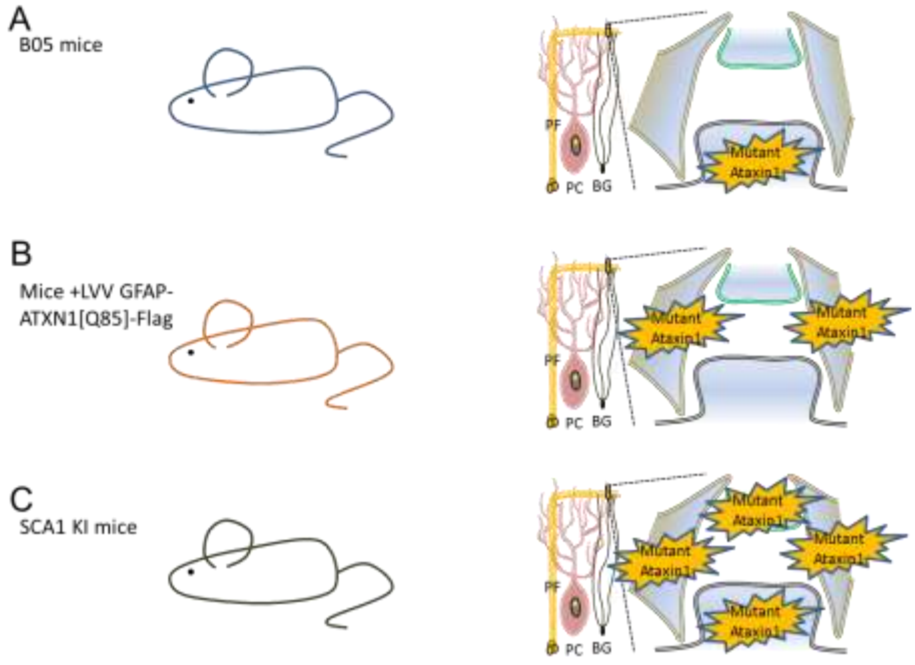

Figure S1. SCA1 models with different distribution of mutant Ataxin-1 in the tripartite PF-PC synapse. (**A**) B05 mice express ATXN1[Q82] selectively in PCs. (**B**) Mice with selective expression of ATXN1[Q85] in BG. (**C**) SCA1 KI mice that express ATXN1[Q154] in a nonselective manner in all units of the tripartite synapse.

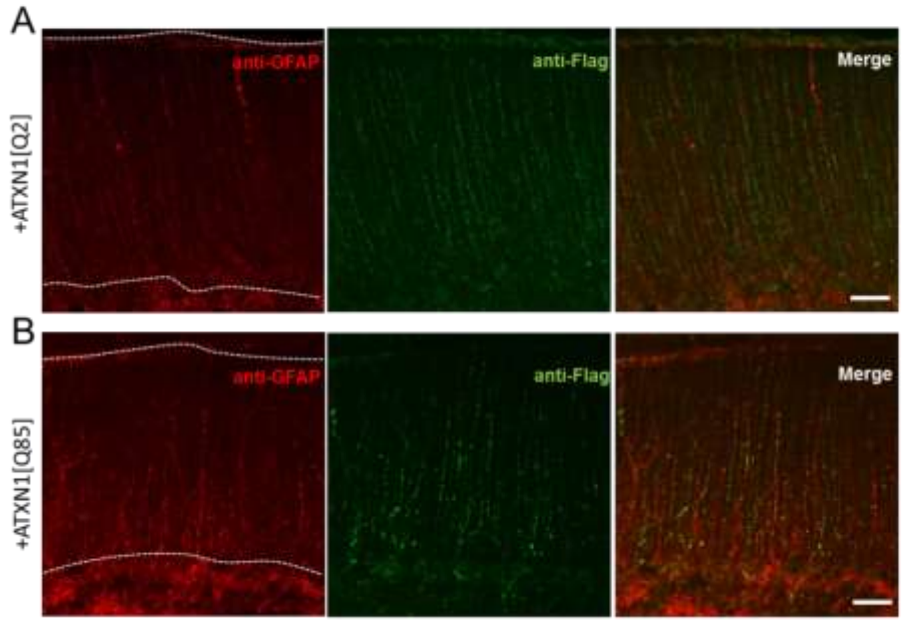

Figure S2. Expression of LVV constructions in BG. Confocal images of cerebellar cortex that express (**A**) ATXN1[Q2] (upper panel) and (**B**) ATXN1[Q85] (lower panel) in processes labeled by anti-GFAP (red) and anti-Flag antibodies (green). ML is marked by dotted lines. Scale bar 50 μm.
